# Bridging Gerontology & the Visual Arts: A Multicomponent Program With Undergraduate Students

**DOI:** 10.1093/geroni/igaf122.411

**Published:** 2025-12-31

**Authors:** Brian Carpenter, Maria Elisa Aguilo

**Affiliations:** Washington University, St. Louis, Missouri, United States; Washington University in St. Louis, St. Louis, Missouri, United States

## Abstract

This session describes a program that bridged gerontology and the visual arts by exposing undergraduate students to older artists and their creative output. The program was one component in a semester-long, introductory interdisciplinary course on aging for first-year students. The program consisted of several elements, including an introductory lecture on creativity across the lifespan; a panel discussion in class with three older professional artists; a visit to a curated museum exhibition featuring works created later in life by Picasso, Miro, Chagall, and Calder, among others; and an assignment that asked students to research a visual artist and reflect on their creativity over their career. A rigorous program evaluation documented the program’s impact on the students. As examples of findings, among the 73 students who participated, their perception of the age at which creativity peaks rose following each component of the program (from 33.8 to 44.5 years old, F(2,59) = 8.59, p < .001), and their rating of the extent to which older adults were “innovative” increased, F(2,60) = 10.82, p < .001. We describe each component of the program, our perceptions of how they shifted student attitudes, and information regarding resources need to replicate the program, in whole or in part. Overall, this program is an innovative melding of gerontology and the visual arts with the potential to challenge ageist beliefs and spotlight the creative potential of older artists.

